# Angelman Syndrome due to familial translocation: unexpected additional results characterized by Microarray-based Comparative Genomic Hybridization

**DOI:** 10.1186/s13039-015-0127-6

**Published:** 2015-04-09

**Authors:** Emiy Yokoyama-Rebollar, Adriana Ruiz-Herrera, Esther Lieberman-Hernández, Victoria Del Castillo-Ruiz, Silvia Sánchez-Sandoval, Silvia M Ávila-Flores, José Luis Castrillo

**Affiliations:** Departamento de Genética Humana, Instituto Nacional de Pediatría, Insurgentes Sur 3700-C. Colonia Insurgentes Cuicuilco. Delegación Coyoacán C.P. 04530. México, D.F., México; Laboratorio de Citogenética, Departamento de Genética Humana, Instituto Nacional de Pediatría, D.F., México; Genetadi Biotech S.L., Bilbao, España

**Keywords:** Angelman Syndrome, Translocation, 15q11.2, array CGH

## Abstract

**Background:**

The 15q11q13 region is subject to imprinting and is involved in various structural rearrangements. Less than 1% of Angelman Syndrome patients are due to translocations involving 15q11q13. These translocations can arise *de novo* or result from the segregation of chromosomes involved in a familial balanced translocation.

**Results:**

A 5-year-old Mexican girl presented with developmental delay, minor dysmorphic features and history of exotropia. G-banding chromosome analysis established the diagnosis of Angelman Syndrome resulting from a familial translocation t(10;15) involving the 15q11.2 region. The available family members were studied using banding and molecular cytogenetic techniques, including Microarray-based Comparative Genomic Hybridization, which revealed additional unexpected results: a coincidental and smaller 15q deletion, asymptomatic duplications in 15q11.2 and Xp22.31 regions.

**Conclusions:**

This report demonstrates the usefulness of array CGH for a detailed characterization of familial translocations, including the detection of submicroscopic copy number variations, which would otherwise be missed by karyotype analysis alone. Our report also expands two molecularly characterized rare patient cohorts: Angelman Syndrome patients due to familial translocations and patients with 15q11.2 duplications of paternal origin.

## Background

Low copy repeats (LCRs) in proximal 15q facilitate recombination events; hence, they are frequently involved in chromosomal structural rearrangements [1]. The 15q11q13 region contains several imprinted genes, such as *UBE3A*, whose *de novo* deletion in the maternal allele causes approximately 70% of Angelman Syndrome (AS) patients and the loss of paternal allele causes Prader-Willi Syndrome (PWS) [2].

On the other hand, unbalanced translocations account for less than 1% of AS patients; these translocations may result from segregation of chromosomes involved in a familial balanced translocation [3]. With the advent of new molecular techniques such as microarray-based Comparative Genomic Hybridization (array CGH), these unbalanced rearrangements can be fully characterized. Furthermore, unexpected chromosomal imbalances have been observed when analyzing complex familial rearrangements, which might affect the phenotype of the involved family members [4,5].

In this report, we present the clinical, cytogenetic and molecular findings of a Mexican patient who fulfills diagnostic criteria established for AS [6], as a result of 3:1 segregation of a familial (10;15) translocation involving 15q11.2. Unexpected additional findings by aCGH in three family members are described in detail.

## Case presentation

**Figure 1:**
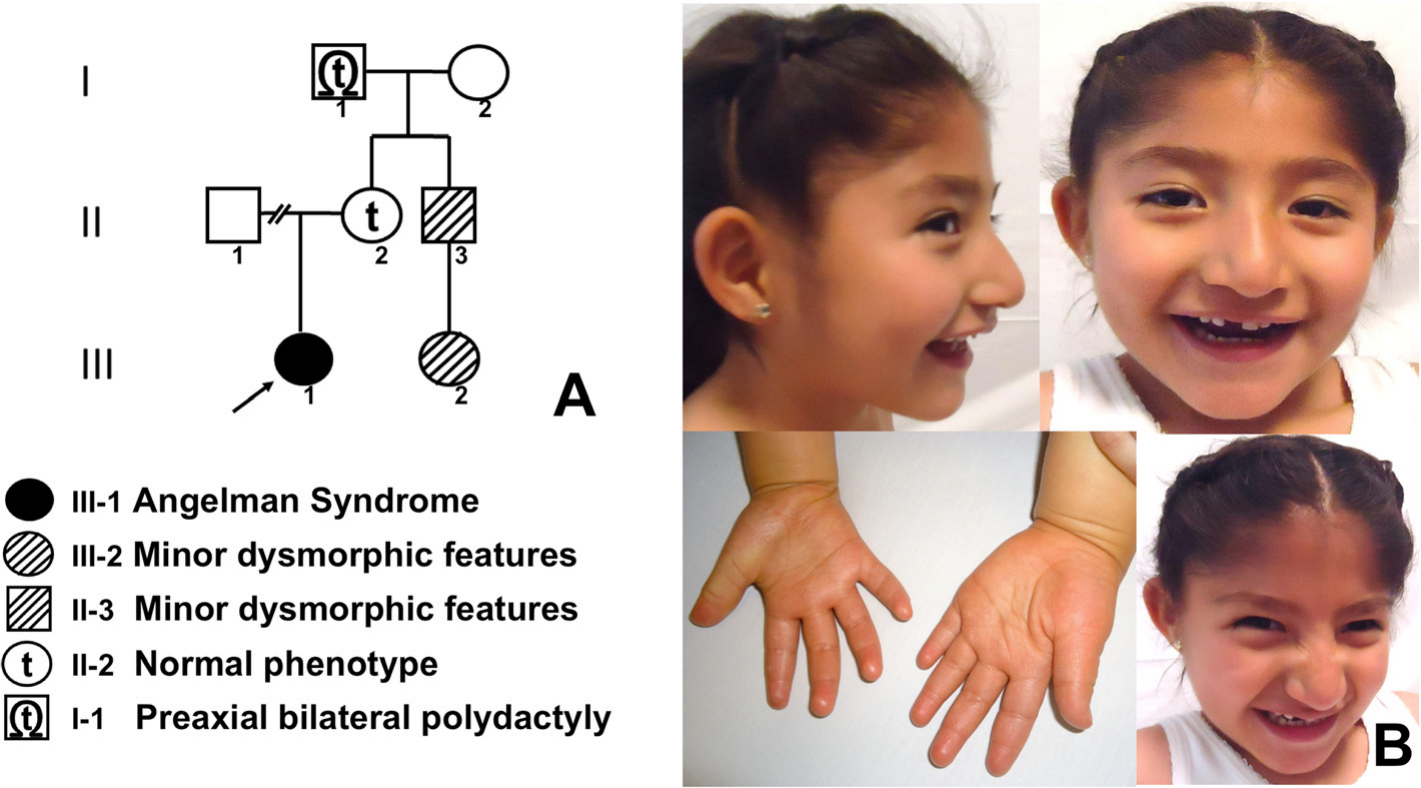
**Available family members. A)** Pedigree of the proband and her family; **B)** Proband (III-1) with telecanthus, bilateral epicanthal folds, wide mouth, and an apparently happy demeanor; hands with tapered fingers, abnormal creases and broad thumbs.

A 5-year-old Mexican girl, first child of a healthy nonconsanguineous couple was evaluated (Figure 1A). She was born at full term via caesarean section, which was indicated because of oligohydramnios detected in the last prenatal ultrasound; otherwise the pregnancy was uneventful. Her birth weight was 2800 g, length was 48 cm; Apgar score of 9.

She was referred to our Medical Genetics service for evaluation because of global developmental delay and a history of exotropia. On physical examination, the weight and height were between the 25^th^ and 50^th^ centile, and the head circumference was in the 10^th^ centile. The patient exhibited slight brachycephaly, low anterior hair implantation, bushy eyebrows, bilateral epicanthal folds, telecanthus, slightly broad nasal bridge, prominent nose with a bulbous tip, short, broad and smooth philtrum, wide mouth, lips with an absent Cupid’s bow, intact palate and uvula, normal pinnae, chest with widely spaced nipples, hands with tapered fingers, broad thumbs and broad 2^nd^ fingers (Figure 1B).

Our patient was considered to have moderate intellectual disability with deficits in all adaptive functions but her language skills were the most affected. At the age of 5, she remained without bowel control and could not run or jump. She climbed stairs with support and only spoke 4 disyllables. In addition, food aversion, excessive salivation, water attraction, fascination with objects that crinkle and squeak (such as certain papers and plastics), constipation and a history of sleep disturbance were also noticed. Her MRI showed mild cortical and subcortical brain atrophy, and her EEG demonstrated paroxysmal activity in the left and right occipital region, which did not generate abnormal movements. While awake, she exhibited multiple movements in both hands that were unrelated to paroxysmal activity.

## Results

**Figure 2:**
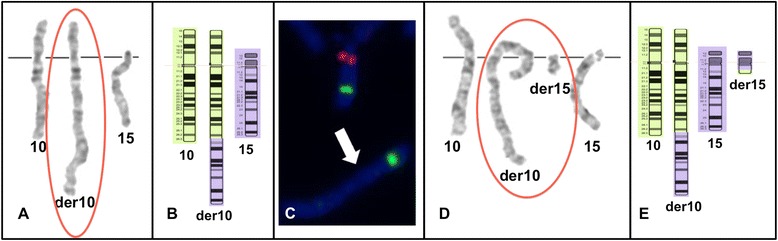
**Cytogenetics results. A)** Partial G-banding karyotype of proband (III-1): 45,XX,der(10)t(10;15)(q26.3;q11.2),-15 [red ovale]; **B)** Ideogram showing normal chromosomes 10 and 15, as well as derivative chromosome 10 of proband (III-1); **C)** FISH analysis of proband (III-1): ish del(15)(q11.2q11.2)(SNRPN-, PMLx2) [SNRP/red, PML/green]; **D)** Partial G-banding karyotype of the proband’s mother (II-2): 46,XX,t(10;15)(q26.3;q11.2) [red ovale]; **E)** Ideogram showing chromosomes 10, 15 and the balanced reciprocal translocation in the proband’s mother (II-2).

**Figure 3:**
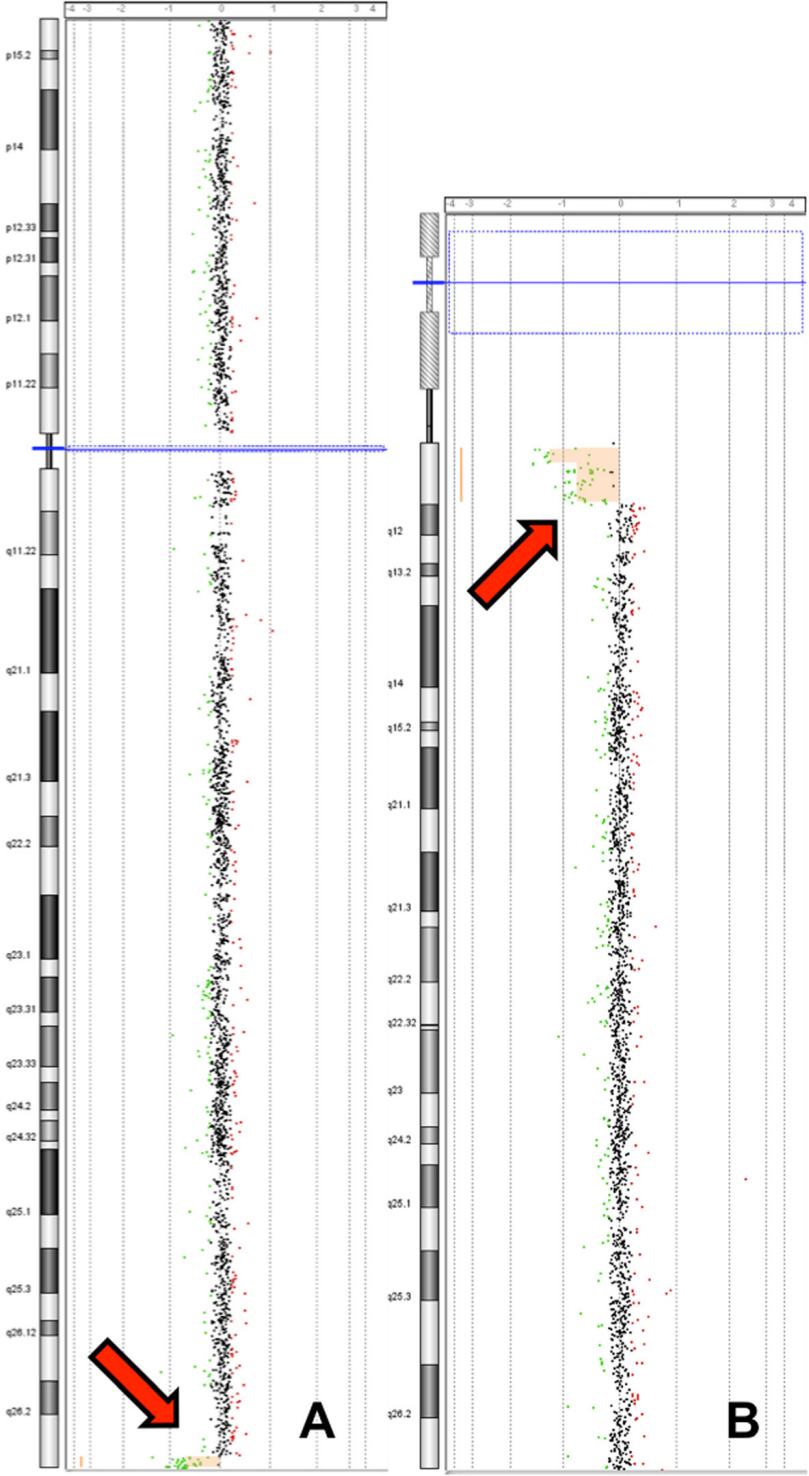
**Array CGH analysis results of the proband (III-1).** 45,XX,der(10)t(10;15)(q26.3;q11.2),-15 mat.arr[hg19] 10q26.3(134,339,232-135,404,471)x1,15q11.1q11.2(20,481,702-25,582,821)x1; **A)** Chromosome 15; **B)** Chromosome 10.

**Figure 4:**
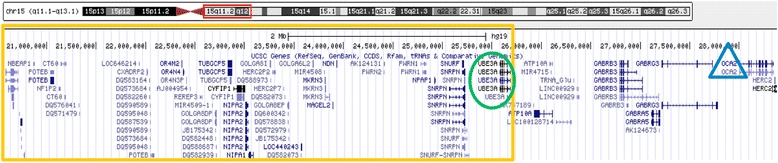
**Gene content of the 15q11.1q11.2 region (yellow rectangle), showing loss of the** ***UBE3A*** **gene (green oval), as well as retention of the** ***OCA2*** **gene (blue triangle).**

G-banding karyotype analysis showed 45,XX,der(10)t(10;15)(q26.3;q11.2),-15 (Figure 2A and B) in the proband (III-1). Subsequently, FISH showed absence of the critical AS/PWS region on the derivative chromosome 10, confirming the diagnosis of AS due to translocation (Figure 2C). Array CGH confirmed that the patient had a 15q11 deletion of 5.1 Mb lacking the *UBE3A* gene, presumably of maternal origin, and a 10q26.3 deletion of 1 Mb (Figures 3 and 4).

**Figure 5:**
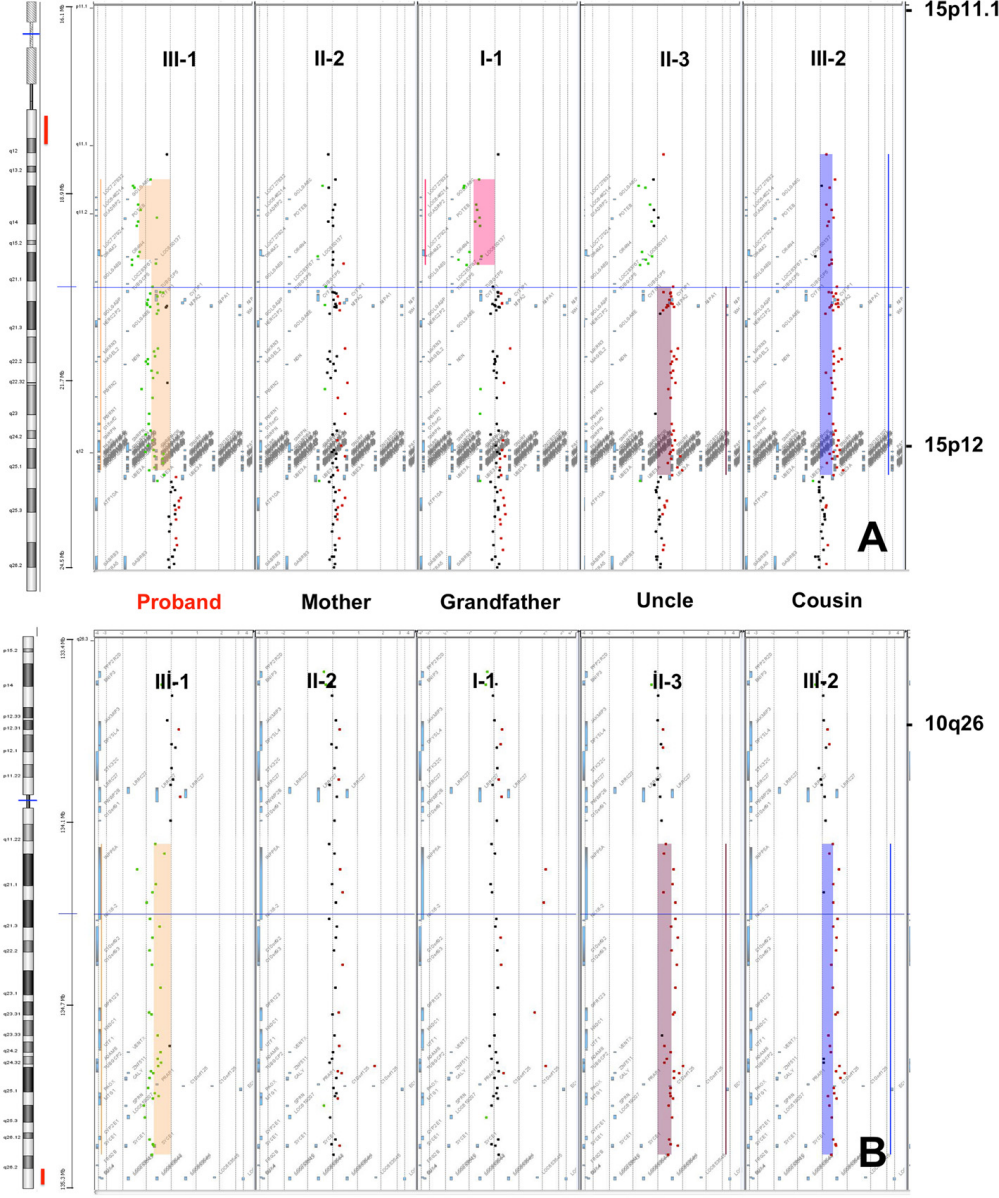
**Array CGH images of the 15q11.1q11.2 region (A) and 10q26.3 region (B) of the five available family members: III-1 (patient), II-2 (mother), I −1 (grandfather), II-3 (uncle), and III-2 (cousin).**

Available family members were also studied (Table 1; Figure 1A); at first, using banding karyotype, the proband’s mother and maternal grandfather were considered balanced carriers of the (10;15) translocation (Figure 2D and E). However, after conducting array CGH, the mother (II-2) was proved to be a truly balanced carrier and the maternal grandfather (I-1), whom only remarkable clinical feature was bilateral preaxial polydactyly, was unexpectedly found to have an additional 2 Mb partial monosomy in the 15q11 region [46,XY,t(10;15)(q26.3;q11.2).arr[hg19] 15q11.1q11.2(20,481,702-22,509,254)x1] (Table 1; Figure 5).

**Table 1:** **Array CGH results in the five available members family**

**Family member**		**Chromosome**	**Imbalanced**	**Position 1**	**Position 2**	**Size**
III-1	Proband	10q26.3	deletion	134,339,232	135,404,471	1.06
		15q11.1-q11.2	deletion	20,481,702	25,582,821	5.1
II-2	Mother	10	normal	---	---	---
		15	normal	---	---	---
I-1	Grandfather	10	normal	---	---	---
		15q11.1-q11.2	deletion	20,481,702	22,509,254	2.03
II-3	Uncle	10q26.3	duplication	134,339,232	135,404,471	1.06
		15q11.2	duplication	22,784,523	25,582,821	2.8
III-2	Cousin	10q26.3	duplication	134,339,232	135,404,471	1.06
		15q11.1-q11.2	duplication	20,481,702	25,582,821	5.1
		Xp22.31	duplication	6,552,712	8,115,153	1.56

Furthermore, banding karyotype in maternal uncle (II-3) and his daughter (III-2) showed a small supernumerary marker chromosome (sSMC) derived from chromosome 15, [47,+der(15)t(10;15)(q26.3;q11.2)] which resulted from a 3:1 segregation of the familial translocation, that consequently led to a partial 15q and partial 10q trisomy. The uncle had a wide nasal base and a wide philtrum, but was otherwise healthy and had normal intelligence. His daughter had a depressed nasal bridge, anteverted nares and tented upper lip vermilion; she reached the expected milestones at 6 months. When the array CGH was performed, they showed a 2.8 Mb partial trisomy 15 in II-3 and a larger 5.1 Mb partial trisomy in his daughter [15q11.2(22,784,523-25,582,821) *vs.* 15q11.1q11.2(20,481,702-25,582,821)] (Table 1; Figure 5).

**Figure 6:**
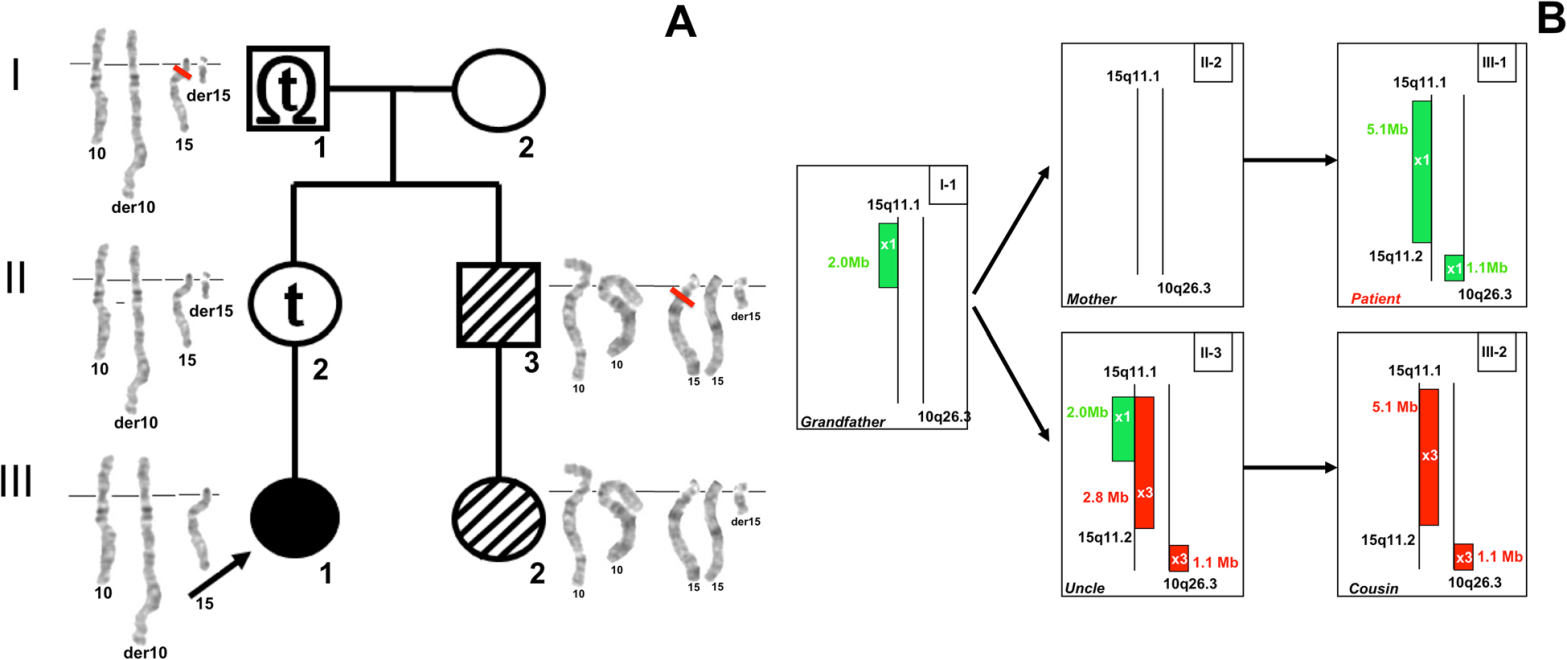
**Familial Chromosomal Segregation. A)** Partial karyotype of each family member showing chromosomes 10, 15 and the translocation; Proband (III-1): 45,XX,der(10)t(10;15)(q26.3;q11.2),-15; mother (II-2): 46,XX,t(10;15)(q26.3;q11.2); grandfather (I-1): 46,XY,t(10;15)(q26.3;q11.2); maternal uncle (II-3): 47,XY,+der(15)t(10;15)(q26.3;q11.2) and his daughter (III-2): 47,XX,+der(15)t(10;15)(q26.3;q11.2). Red rectangle shows the 15q11 microdeletion in the grandfather and maternal uncle. **B)** Schematic representation of array CGH results of 15q11.1q11.2 and 10q26.3 chromosomal regions in the five available family members. The deletions (x1, green), and duplications (x3, red) are shown as rectangular boxes on the left or the right of the vertical chromosomic lines, respectively. The chromosomal imbalance between the maternal uncle (II-3) and his daughter (III-2) can be explained, because he had the same deletion in the non-translocated chromosome 15, as his father (I-1).

These findings led us to suspect that the grandfather’s unexpected microdeletion was in the non-translocated chromosome 15 (Figure 6), confirmed by locus-specific FISH probes, and we inferred that the maternal uncle (II-3) had the same chromosomal 15 microdeletion, this could not be confirmed because he denied to provide more blood sample. Moreover, the chromosomal imbalance between the uncle and his daughter (III-2) can be explained by all these findings and also by the segregation in her, of the paternal non-deleted chromosome 15 (Figure 6). Lastly, she had an Xp22.31 duplication of 1.66 Mb, additionally to the 15-derived sSMC, detected by array CGH analysis (Table 1).

## Discussion

Cases of Angelman syndrome resulting from familial translocations are rare (less than 1%) [3]. Due to imprinting, the family described in the present report is at risk for two different neurogenetic disorders, AS and PWS, which are rarely seen together in the same kindred [7,8]. The proband in this family has a 5 Mb partial monosomy of the 15pterq11.2 region and a 1 Mb partial monosomy of 10q26.3qter as a result of 3:1 segregation of a familial translocation (10;15), which is the most frequently observed when an acrocentric chromosome is involved [9,10]. The partial deletion in our patient at chromosome 15 is of maternal origin, and was proved to include the *UBE3A* gene leading to the diagnosis of AS [11].

When she was evaluated, some of her features were not in accordance with classical AS patients, because her developmental delay was functionally moderate and not severe, in addition she did not have marked ataxia or laughter. Yet, she fulfilled the diagnostic criteria for AS given that she exhibited unsteadiness and clumsiness, speech impairment with minimal use of words, and the EEG abnormalities among other features already described [6]. Partial monosomy of distal 10q has been associated with craniofacial, cardiac, and urogenital defects, as well as with neuropsychiatric disorders [12]; however, the deletion in our patient is distal to the critical region for this phenotype. Therefore, we can attribute the proband’s phenotype to partial 15q monosomy.

The region involved in the maternal uncle (II-3) and his daughter (III-2) is outside from the critical region described in the10q distal trisomy syndrome. This led us to expect them to have a normal phenotype [13]. It is important to point out that their triple dose of 15q11q11.2 is of paternal origin, when most of the reports with abnormal phenotype due to a sSMC involving the critical region for PWS/AS, are primarily of maternal origin [14]. Michelson *et al.* described a carrier of a 15-derived supernumerary chromosome of maternal origin. This patient had macrocephaly, ventricular dilatation, hypotonia, epilepsy and intellectual disability [15]. Other reports of 15q11q13 trisomy or tetrasomy have attributed the phenotype to the maternally expressed genes dosage including *UBE3A*, as well as to the non-imprinted genes, such as *GABA receptor subunit* gene, which are involved in epileptogenesis [16-18]. There are few reports of interstitial 15q11q13 paternal origin duplications associated with abnormal phenotypes [19,20].

The 15q11.1q11.2 2 Mb-deleted region in the grandfather (I-1) and maternal uncle (II-3) contains only 15 genes, of which 10 are non-coding RNAs, 3 are mRNA with unknown function and 2 encode for olfactory receptors; therefore we believe that this microdeletion does not alter their phenotype. It is important to recall that the 15q11q13 region is flanked by 5 breakpoints (BPs), and typical AS/PWS deletions have BP1 or BP2 as the proximal breakpoint and BP3 as the distal breakpoint. Recently, a microdeletion between BP1 and BP2 has been associated to different phenotypes [21-24]; however, after conducting research of published reports and databases such as DECIPHER, we did not find any patient reported to have the same distal imbalance proximal to BP1 as the grandfather has (I-1).

Regarding the 1.56 Mb microduplication of the Xp22.31 region observed in the proband’s cousin (III-2), there have been at least 35 patients described with this same duplication, its pathogenicity remains controversial [25]. A previous study reported the prevalence of the microduplication to be 0.15% in healthy individuals and 0.37% in patients with intellectual disability or behavioral disturbances, suggesting a modifier or risk factor for disease; however, this result was not statistically significant [26]. Another study also indicated that this condition might predispose to an abnormal phenotype; nevertheless, the authors stated that additional genomic changes are required [27].

Reports of familial translocations involving 15q11q13 [7,14,28-34] including ours, have shown the importance of karyotype analysis as part of the diagnostic approach in PWS and AS patients, especially in those where the phenotype is not classical. This analysis should be performed in order to search for underestimated structural chromosomal rearrangements that could be inherited, with important implications in the recurrence risk and the possibility of prenatal diagnosis [7,35]. In fact, some diagnostic algorithms for PWS/AS already include the use of karyotype [36].

## Conclusion

The present report is another example of the importance of molecular characterization using array CGH in familial translocations to accurately define genomic imbalances for each family member, as well as for detection of submicroscopic copy number variations, which would otherwise be missed by karyotyping alone. The detailed clinical, cytogenetic and molecular characterization also contributes to pursue a genotype phenotype correlation.

## Methods

G-banding cytogenetic studies were performed from peripheral blood lymphocytes by standard method of GTG banding technique (Giemsa). Subsequently, genomic DNA was obtained from all family members in whom karyotype revealed a translocation (10;15) or the presence of any derivative of this rearrangement. Whole-genome array CGH analysis was performed using 500 ng of genomic DNA and a 60 K oligonucleotide array (Agilent Technologies, Santa Clara, CA, USA; design G4450A) according to protocols provided by the manufacturer. Image quantification, hybridization quality control and copy number variants (CNVs) detection were performed using Agilent Feature Extraction v11.5 and Agilent Workbench v7.0. CNVs identified in the samples were visualized using the UCSC Genome Browser website (http://genome.ucsc.edu) and compared to the Database of Genomic Variants (http://projects.tcag.ca/variation) to exclude copy number changes considered to be benign variants. The DECIPHER (Database of Chromosomal Imbalance and Phenotype in Humans using Ensembl Resources) (https://decipher.sanger.ac.uk/) and ECARUCA (European Cytogeneticists Association Register of Unbalanced Chromosome Aberrations) (http://umcecaruca01.extern.umcn.nl:8080/ecaruca/ecaruca.jsp) databases were used as resources to aid in the genotype-phenotype correlation. Validation of variants detected by array CGH was performed by fluorescence *in situ* hybridization (FISH) using Kreatech probes (http://www.kreatech.com/) with the standard methodology.

## Consent

Written informed consent was obtained from the patient’s parents for publication and accompanying images of this case report. A copy of the written consent is available for review by the Editor-in-Chief of this journal.
